# A Moderated Mediation Model to Predict Adolescent Resistance to Peer Influence: Evidence From an Adoption Study

**DOI:** 10.1002/jad.70016

**Published:** 2025-07-13

**Authors:** Li (Hazel) Yu, Kristine Marceau, Valerie S. Knopik, Misaki N. Natsuaki, Daniel D. Shaw, Leslie D. Leve, Jody M. Ganiban, Jenae M. Neiderhiser

**Affiliations:** ^1^ Department of Human Development and Family Science Purdue University West Lafayette IN USA; ^2^ Department of Psychology University of California Riverside CA USA; ^3^ Department of Psychology University of Pittsburgh Pittsburgh PA USA; ^4^ Prevention Science Institute University of Oregon Eugene OR USA; ^5^ Department of Psychological and Brain Sciences George Washington University Washington DC USA; ^6^ Department of Psychology Penn State University University Park PA USA

**Keywords:** adolescence, adoption design, gene‐environment interaction, impulsivity, resistance to peer influence, self‐esteem

## Abstract

**Introduction:**

Adolescents are particularly susceptible to peer influence and at higher risk of engaging in problematic behaviors through peer interactions, but also vary in the extent to which they are influenced by their peers. Resistance to peer influence, the tendency to refuse undesired peer pressure, is one key factor for this variation. However, how genetic and contextual influences shape the development of RPI remain unclear.

**Methods:**

The present study leveraged data from 552 family triads, collected in the U.S. between 2003 and 2022, in an adopted‐at‐birth design. Family triads included an adopted child (57.2% male; 55.3% European American, 19.6% multiracial, 13.2% African American, 10.9% Hispanic/Latinx), birth parents, and adoptive parents. Moderated mediation models were tested to examine whether: (1) child phenotypic impulsivity at age 7 mediated the association between genetic risk for impulsivity and adolescent RPI; (2) child phenotypic self‐esteem (age 6–8) mediated the association between genetic factor for self‐esteem and adolescent RPI; (3) adoptive parent responsiveness weakened the impulsivity pathway; and (4) adoptive parent responsiveness strengthened the self‐esteem pathway. Analyses were performed separately for age 11 (early adolescent; *M*_age_ = 11.39, SD_age_ = 0.55) and age 13–15 (middle adolescent; *M*_age_ = 14.97, SD_age_ = 1.24) RPI as the outcome variable.

**Results:**

Results revealed that birth parent self‐esteem positively predicted early adolescent RPI, through elevated levels of child self‐esteem. RPI was moderately stable from early to middle adolescence.

**Conclusions:**

Findings highlight the importance of nurturing children's self‐esteem to improve their skills to resist undesired peer pressure during adolescence.

## 1 Introduction

Adolescence is a developmental stage characterized by both increased exposure to peer contexts and stronger peer influences (Brechwald and Prinstein 2011; Brown et al. 1997). Both survey and experimental studies indicate that problematic behaviors (e.g., underage drinking, binge drinking, bullying) can be contagious within peer networks through direct encouragement, imitation, and peer norms and pressure (e.g., DiGuiseppi et al. 2018). Resistance to peer influence (RPI), a socio‐cognitive skill that indexes psychosocial maturation (Bell and Baron 2015; Sumter et al. 2009), acts as a potential protective factor in developmental trajectories of problematic behaviors incented by peers. Defined as the tendency to reject undesired peer norms and pressure (Steinberg and Monahan 2007), RPI has been found to buffer the influence of perceived peer norms on binge drinking in college students, risky sexual behavior in mid‐adolescence (age 14–15 years), and antisocial behavior in mid‐to‐late adolescence (14–18 years) (Choukas‐Bradley et al. 2015; DiGuiseppi et al. 2018; Teunissen et al. 2016; Wojciechowski 2018). The current study investigated the development of RPI to elucidate risk and protective processes that support or hinder the development of this competency (O'Connell et al. 2009).

### 1.1 Resistance to Peer Influence (RPI) in Adolescence

Previous studies showed that RPI gradually develops in early‐to‐mid adolescence, and increases rapidly in middle‐to‐late adolescence, with girls reporting higher levels of RPI than boys (Steinberg and Monahan 2007; Sumter et al. 2009). In addition to protective effects cited above, RPI has been found to decrease involvement in antisocial activities (Walters 2018), and buffer the influence of affiliating with antisocial peers (Monahan et al. 2009; Wojciechowski 2018). Overall, the literature indicates that a higher level of RPI is a developmental competency that plays a protective role in the association between peer norms with adolescent substance use and deviant behaviors.

### 1.2 The Mediating Roles of Child Phenotypic Impulsivity and Self‐Esteem

Several individual characteristics related to impulsivity and self‐esteem have emerged as key predictors of RPI. These constructs are distinct, and typically uncorrelated or modestly (*r* = −0.12 to 0.09; Davenport et al. 2012; Miyadera 2024), but are both shaped by genetic influences, parenting styles (Pinquart and Gerke 2019; Ran et al. 2021), and quality of the home environment (Mulligan et al. 2013; Orth et al. 2018)—albeit typically in opposite directions. Thus, these phenotypes are conceptualized as two independent pathways that may shape the development of RPI.

Impulsivity is defined as a tendency to act without planning or reflecting on the consequences of actions (Moeller et al. 2001). Self‐esteem, as a key form of self‐cognition, has been defined as individuals' subjective evaluation of their worth as a person (Orth and Robins 2014). A cross‐sectional study on British adolescents (16–18 years) suggested that those who are high in negative urgency (an aspect of emotional impulsivity) reported lower RPI (Stautz and Cooper 2014). Research focusing on Mexican American mid‐to‐late adolescents and undergraduate students in China found that self‐esteem was positively associated with RPI (Bámaca and Umaña‐Taylor 2006; Chen et al. 2016). Another cross‐sectional study on Canadian mid‐adolescents found that high self‐esteem was associated with lower susceptibility toward negative peer influence (e.g., a phenotype closely related to RPI; Yang et al. 2013).

Impulsivity and self‐esteem are heritable (e.g., based on twin and molecular genetic studies, impulsivity: Elam et al. 2017; Niv et al. 2012; self‐esteem: Cai and Luo 2017; Raevuori et al. 2007) and influenced by family environments, such as parent‐child interactions (Zhang et al. 2020), mother‐child attachment (Olson et al. 1990), and parenting styles (Pinquart and Gerke 2019). However, the contributions of genetic and familial environmental factors on RPI have been understudied, a gap we begin to fill in the current study. Although there is a lack of direct evidence indicating the heritability of RPI, RPI has been regarded as an indicator of autonomy (Monahan et al. 2009), which is moderately heritable (e.g., heritability = 41%; Loehlin et al. 2003) and shares genetic influences with both impulsivity and self‐esteem (e.g., Malanchini et al. 2019; Saphire‐Bernstein et al. 2011). Therefore, we posited that RPI would be predicted by genetic factors of impulsivity and self‐esteem and perhaps mediated through the adolescents' impulsivity and self‐esteem phenotypes, respectively.

Furthermore, previous evidence shows that impulsivity relies on cognitive skills, including motor inhibition, delayed discounting of reward, and decision making (e.g., Bickel et al. 1999; Lejuez et al. 2002), neurological processes that also underlie the ability to resist peer influence (Grosbras et al. 2007; Pei et al. 2020). Impulsive children may find it hard to acquire mature decision‐making skills, tend less to reflect before taking actions and inhibit the tendency to follow the norms set by peers, and thus be less likely to resist peer influences. Mechanistically, this mediation pathway would be explained in part through shared genetic influences and a developmental trajectory where impulsivity‐related genetic influences lead to increased impulsivity (possibly via delayed maturation of neurological processes related to inhibiting impulsive choice) that then hampers youths' development of RPI.

Adolescents with higher self‐esteem are more likely to refer to their own beliefs and values to make decisions rather than relying on others, and thus may be more likely to resist peer influence. Neuropsychological evidence has found that self‐esteem is associated with dorsal anterior cingulate cortex and anterior insula activation (Eisenberger et al. 2011; Izuma et al. 2018), brain regions that are found to also influence RPI and to be influenced by genetic factors (Way et al. 2009). The high overlap between phenotypic and neuropsychological levels of self‐esteem and RPI suggests that genetic and/or environmental factors related to low self‐esteem may alter the development of neurological functioning (e.g., rejection sensitivity, decision making) (Way et al. 2009), which further leads to lower levels of self‐esteem over childhood and RPI during adolescence.

### 1.3 The Moderating Role of Parental Responsiveness

Empirical work focusing on identifying familial factors that maintain, amplify, or reduce developmental outcomes among infants, children, and adolescents with high impulsivity and low self‐esteem has been well established (e.g., Bulanda and Majumdar 2009; Herz and Gullone 1999; Menting et al. 2016). Experiencing more positive parenting (e.g., high‐quality guidance and support) can buffer children with high impulsivity from developing aggressive and antisocial behaviors during adolescence (Cao et al. 2024; Giannotta and Rydell 2016). High levels of parental care and acceptance have also been shown to alleviate the influence of the Dopamine Transporter (*DAT1*) gene (i.e., one key genetic risk for impulsivity) in children (e.g., 6–11 years; Marzilli et al. 2023). Negative family environments, such as family conflict, exacerbated the association of polygenic risk for alcohol use disorder (used as a proxy for genetic risk on impulsivity given that impulsivity is a key predictor of alcohol use) on impulsivity in children (e.g., 9–10 years; Su et al. 2022). Research directly investigating the moderating role of parental responsiveness in the effects of genetic risk for impulsivity and on self‐esteem, however, remains scarce.

Parental responsiveness involves warm acceptance of the child's needs and distress, and sensitive and contingent responses toward child signals (Ainsworth et al. 2015; Davidov and Grusec 2006; Merz et al. 2017). Positive parenting practices can nurture children's self‐control skills such as delayed discounting through providing appropriate feedback, encouragement, and guidance (Molina and Musich 2016; Scaramella and Leve 2004), which is also supported by experimental intervention studies, including the Family Check‐Up (Chang et al. 2014, 2015, 2017). Growing up in a family with high levels of emotional and instrumental support would alleviate the association between genetic factors for impulsivity and child phenotypic impulsivity (Paaver et al. 2008; Propper and Moore 2006).

Similarly, parental responsiveness has been shown to play a crucial role in moderating the development of self‐esteem (Blattner et al. 2013). Highly responsive parents are more likely to initiate and engage in open and supportive parent–child communications (Blattner et al. 2013). During communication, parents and children collaborate on life experiences, promote meaning‐making, and parents provide constructive feedback and effective solutions to children's concerns (Marin et al. 2008; Reese et al. 2007). Children who show confident characteristics in early years due to inherited genetic factors, if exposed to higher level of open communication and high‐quality reinforcement from parents, may be more likely to develop consistently high self‐esteem.

### 1.4 Present Study

The present study aimed to address four research questions: (1) Does child phenotypic impulsivity mediate genetic risks for adolescent RPI? (2) Does child phenotypic self‐esteem mediate genetic factors for adolescent RPI? (3) Does parental responsiveness moderate the influences of genetic risks on impulsivity? (4) Does parental responsiveness moderate the influences of genetic factors on self‐esteem?

A strength of the current study is the inclusion of gene‐environment interplay, which can avoid misattributing the causes of RPI solely to either genetic or environmental factors, thereby improving the robustness of conclusions. Specifically, we used an adopted‐at‐birth design to elucidate the influence of genetic and family factors on RPI respectively, and to rule out the potential that parental responsiveness is affected by adolescents' heritable predispositions towards impulsivity and/or self‐esteem that biological parents share with their biological child (i.e., passive gene‐environment correlation; *r*GE). In this adopted‐at‐birth design, adopted children were placed in families unrelated to their birth parents, and thus their birth parent only contributed genetic and prenatal factors to child phenotypes. Therefore, associations between birth parents' and the adopted children's phenotypes are explained by their shared genetic makeup. Since adoptive parents do not share genes with their adopted child, the impacts of parental responsiveness on children's phenotypes reflect purely environmental influences, or evocative processes (i.e., the child's genetically influenced traits evoke specific responses from their adoptive parents) of certain genetic factors in the child.

Based on previous research, we hypothesized that: (1) heritable predispositions toward RPI are associated with adolescent RPI at age 11 and 13–15 (see “Measures” for more details) via child impulsivity; (2) heritable predispositions toward RPI are associated with adolescent RPI at age 11 and 13–15 via child self‐esteem; (3) adoptive parent responsiveness attenuates the relations between the genetic predispositions of birth parent impulsivity and child impulsivity; and (4) adoptive parent responsiveness strengthens the association between the genetic predispositions of birth parent self‐esteem and child self‐esteem.

## 2 Methods

### 2.1 Participants

Data for the present study were drawn from the Early Growth and Development Study (EGDS), which included 561 family triads (e.g., the child who was adopted at birth, adoptive parents, and birth parents) recruited from 45 adoption agencies in 15 states in the United States (Leve et al. 2019, 2007). Families were recruited in two cohorts and with data available from birth to age 15 (Cohort I) or 13 (Cohort II) at the time of this study. The adopted children were 57.2% male, and 55.3% European American, 19.6% multiracial, 13.2% African American, 10.9% Hispanic or Latinx, and < 1% each for Asian, Pacific Islander, American Indian and unknown ethnicity. The majority of adoptive parents were in middle to upper socioeconomic status, with a median annual household income above $100k. More than 70% of adoptive parents had completed a college education or had advanced to graduate school for an additional degree. 33.1% of birth mothers and 40.7% of birth fathers had a high school degree. Birth mothers had a median annual household income of less than $15,000 and birth fathers between $15,000 and $25,000.

### 2.2 Procedures

Recruitment began in March 2003, with the recruitment of adoption agencies in the US (Leve et al. 2019, 2007). Eligibility criteria included: (1) the adoption placement was domestic, (2) placement occurred within 3 months after birth, (3) the adoptive family was not biologically related to the adopted child; (4) the adopted child did not experience any major medical conditions, and (5) both the birth and adoptive parents could understand English as the 8th grade level. Family triads satisfying these eligibility criteria were invited to participate and signed consent forms. In‐home interviews, phone interviews, online surveys, and written surveys were conducted according to the requirements of measures and waves of data collection. All procedures and assessments were approved for the EGDS by the Institutional Review Board (IRB) of collaborating institutions: University of Oregon (IRB#04262013.034; IRB#03042014.001; IRB#04262013.035; IRB#08082016.007), George Washington University (IRB#041119), Penn State University (STUDY00007603), and Purdue University (IRB‐2022‐522).

### 2.3 Measures

#### 2.3.1 Birth Parent Measures Indexing Genetic Influences

##### 2.3.1.1 Impulsivity

Birth parent impulsivity was assessed as a composite measure, consistent with prior work in EGDS (Harold et al. 2013). Birth parents completed the Adult Temperament Questionnaire – Inhibitory Control Subscale (Derryberry and Rothbart 1988; Evans and Rothbart 2007), when the child was aged 18 months. Birth mother (BM) and father (BF) reported on a 7‐item, 7‐point scale (*r*_BM, BF_ = 0.07; *α*_BM_ = 0.41, *α*_BF_ = 0.30). Birth parents also completed the 9‐item Barkley's Adult ADHD Scales – Hyperactivity/Impulsivity Subscale (Murphy and Adler 2004) when the child was 4.5 years of age, on a 4‐point scale (*r*_BM, BF_ = 0.04; *α*_BM_ = 0.80, *α*_BF_ = 0.71). Despite a modest correlation (*r* = 0.21), the scores were then standardized and averaged to create a final composite score that purposefully encompasses a broader definition of birth parent's impulsivity (Harold et al. 2013).

##### 2.3.1.2 Self‐Esteem

The Harter Adult Self‐Perception Profile—Global Self‐Worth 6‐item subscale (Harter 2015; Messer and Harter 1986) was used to measure birth parent self‐esteem, at 3–6 months post‐partum. Birth parents were asked to select which of a pair of statements best described them, and whether the statement was “Really true for me” or “Sort of true for me” yielding a 4‐point scale for each item. Responses were averaged across items and across both birth parents (*r*_BM, BF_ = 0.23; *α*_BM_ = 0.85, *α*_BF_ = 0.83).

#### 2.3.2 Adoptive Parent

##### 2.3.2.1 Parental Responsiveness

The Home Observation for Measurement of the Environment (HOME), completed during an in‐home interview, was used to assess adoptive mother (AM) and adoptive father (AF) responsiveness when the child was 9, 18, and 27 months of age (Caldwell and Darling 1999). Interviewers rated each AM and/or AF on 10 items (yes/no) reflecting their emotional and verbal responsivity toward the child during the home visit. Scores were averaged across three timepoints and then across both caregivers (*r*_AM, AF_ = 0.50; *α*_AM_ = 0.54, *α*_AF_ = 0.63).

#### 2.3.3 Adopted Child/Adolescent

##### 2.3.3.1 Impulsivity

The Children's Behavior Questionnaire (CBQ)—Impulsivity subscale was used to measure children's impulsivity at 7 years old (Putnam and Rothbart 2006; Rothbart et al. 2001). Adoptive mothers and fathers reported on 13 items focusing on the child's speed and performance of response initiation on a 7‐point Likert scale. Scores were across items (*α*_AM_ = 0.82, *α*_AF_ = 0.77) and across both caregivers' reports (*r*_AM, AF_ = 0.59).

##### 2.3.3.2 Self‐Esteem

Thirteen items from the Junior Temperament and Character Inventory were selected to assess children's self‐esteem (Luby et al. 1999), when the child aged 8 (Cohort I) or 6 (Cohort II). The selection of items was based on prior self‐esteem assessments for children specifically, such as the Behavioral Rating Scale of Presented Self‐Esteem in Young Children (Haltiwanger 1989) and the Coopersmith Self‐Esteem Inventory (Ryden 1978). Example items included “Doesn't know what to do when faced with a problem. (reverse‐coded)”, “Generally sets goals and follows them (attain new skills, good grades, meet new people)”. Adoptive parents reported on a binary scale (1 = True, 0 = False). Scores were converted to pro‐rated sum (*α*_AM_ = 0.79, *α*_AF_ = 0.79), and then averaged across AM and AF reports (*r*_AM, AF_ = 0.45).

##### 2.3.3.3 Resistance to Peer Influence

The Resistance to Peer Influence Questionnaire (Steinberg and Monahan 2007) was used to assess adolescent RPI. RPI was first measured in early adolescence, at age 11 for both cohorts; and then in mid‐adolescence, which was at age 15 for Cohort I and age 13 for Cohort II which were combined to create an age 13–15 RPI score. This questionnaire includes seven pairs of statements. Adolescents select which of a pair of statements best described them, and whether the statement was “Really true for me” or “Sort of true for me” yielding a 4‐point scale for each item that were then averaged (*α*_Age11_ = 0.55, α_Age13–15_ = 0.56).

#### 2.3.4 Covariates

Covariates in the present study included (1) adolescent sex assigned at birth, (2) adolescent age at the RPI assessment, (3) openness in the adoption between birth and adoptive families, and (4) obstetric complications during birth mother's pregnancy (Marceau et al. 2016). It is important to control for adolescent age and sex, because previous evidence shows that the level of RPI increases during adolescence, and girls usually manifest higher RPI than boys (Steinberg and Monahan 2007). Openness in the adoption was included as a covariate to account for contact and communication between birth family and adoptive family, which may lead birth family to influence postnatal environments of the adopted child (Ge et al. 2008). Obstetric complications show associations with infant and child impulsivity (e.g., Navalón et al. 2022) and may confound the estimates of genetic and environmental influences.

### 2.4 Missing Data

During data cleaning, cases missing all main variables were removed. The final sample for analysis consisted of 552 family triads. There was substantial missingness of child impulsivity (37.68%), self‐esteem (29.53%), and RPI variables (32.07% at age 11, 51.99% at age 13–15; see Table 1), Little's Missing Completely at Random (MCAR) test suggested that the missingness of these three variables was completely at random (*χ* ^2^ = 22.8, *df* = 28, *p* = 0.75) (Little 1988). For hypothesis testing, a full information maximum likelihood (ML) estimator was used to accommodate missing data.

**Table 1:** Sample size (*N*), mean (*M*), standard deviation (SD), skewness, and kurtosis results of main variables.

Role	Variable	*N*	Mean	SD	Skewness	Kurtosis	Assessment Timing (In AC Age)
BP	Impulsivity (ATQ; self‐report)	462	3.85	0.78	−0.09	0.36	1.30–2.64 (*M*_*age*_ = 1.51)
	Impulsivity (Barkley's; self‐report)	518	6.25	3.99	1.00	0.92	1.98–3.73 (*M*_*age*_ = 2.29)
	Impulsivity composite score	540	−0.00	0.84	0.58	1.04	/
	Self‐esteem (self‐report)	548	2.93	0.70	−0.49	−0.25	0.54–1.55 (*M*_*age*_ = 0.78)
AP	Responsiveness (observation)	552	10.39	0.70	−1.57	2.18	/
AC	Age 6/8 Impulsivity (AP report)	344	4.62	0.69	−0.23	−0.19	Cohort I: 6.76–8.85 (*M*_*age*_ = 7.07) Cohort II: 6.00–8.00 (*M*_*age*_ = 6.13)
	Age 7 Self‐esteem (AP report)	389	8.60	2.84	−0.50	−0.43	6.00–9.00 (*M*_*age*_ = 7.37)
	Age 11 RPI (self‐report)	375	3.00	0.49	−0.32	−0.13	10.48–13.54 (*M*_*age*_ = 11.39)
	Age 13–15 RPI (self‐report)	265	2.93	0.47	−0.09	−0.09	12.68–17.94 (*M*_*age*_ = 14.97)

*Note:* The impulsivity composite score was created by averaging the standardized scores of ATQ and Barkley's. The AP responsiveness score was created by averaging the scores measured in Wave A (child age range = 0.54–1.55, *M*_*age*_ = 0.78), B (child age range = 1.30–2.64, *M*_*age*_ = 1.51), and C (child age range = 1.98–3.73, *M*_*age*_ = 2.29). Because the distribution of AP responsiveness (skewness = −2.07) was skewed, the outliers were then winsorized. The new skewness value after winsorization is showed in the table.

Abbreviations: AC = adopted child, AP = adoptive parent, ATQ = adult temperament questionnaire, BP = birth parent, Barkley's = Barkley's Adult ADHD Subscales.

### 2.5 Analytic Strategy

Structural equation modeling (SEM) implemented in Mplus 8 was applied to test hypotheses, using bootstrapped confidence intervals with 5000 resamples, as recommended by Preacher and Hayes (2008). After assumption checks, child impulsivity and self‐esteem entered the model as a mediator to the association between birth parent impulsivity/self‐esteem and adolescent RPI. Birth parent impulsivity and self‐esteem were indices of genetic factors for these two phenotypes respectively. Adoptive parent responsiveness was a moderator for both birth parent impulsivity → child impulsivity and birth parent self‐esteem → child self‐esteem pathways. Adolescent age, sex, parental openness to adoption, birth mother obstetric complications were included as covariates. Model fit was evaluated using chi‐square statistics, the Comparative Fit Index (CFI), Tucker‐Lewis Index (TLI), and Root Mean Square Error of Approximation (RMSEA) (Schermelleh‐Engel et al. 2003).

## 3 Results

### 3.1 Descriptive Statistics

The sample size, mean, standard deviation, skewness, and kurtosis for each variable are shown in Table 1.

### 3.2 Correlation Results

Pearson bivariate correlations are shown in Table 2. Self‐esteem and impulsivity composite score were negatively correlated for BPs (*r* = −0.23, *p* < 0.001), and for adopted children (*r* = −0.12, *p* = 0.06). BP's self‐esteem was positively correlated with adopted child self‐esteem (*r* = 0.11, *p* = 0.04), and negatively correlated with adopted child impulsivity (*r* = −0.13, *p* = 0.02), indicating genetic influence, albeit both in small magnitudes. Adoptive parent's responsiveness was positively related to child self‐esteem (*r* = 0.14, *p* = 0.01), indicating environmental influence. Child RPI was moderately correlated from age 11 to age 13–15 (*r* = 0.25, *p* < 0.001).

**Table 2:** Bivariate correlation results of main variables.

	1 BP Impulsivity (ATQ)	2 BP Impulsivity (Barkley's)	3 BP Impulsivity (composite)	4 BP Self‐esteem	5 AP Responsiveness	6 AC Impulsivity	7 AC Self‐esteem	8 AC Age 11 RPI	8 AC Age 13–15 RPI
1 BP Impulsivity (ATQ)	1								
2 BP Impulsivity (Barkley's)	0.21***	1							
3 BP Impulsivity (composite)	0.80***	0.82***	1						
4 BP Self‐esteem	−0.16***	−0.20***	−0.23***	1					
5 AP Responsiveness	0.05	0.00	0.04	0.08+	1				
6 AC Impulsivity	−0.00	0.10+	0.08	−0.13*	−0.09	1			
7 AC Self‐esteem	−0.10	0.00	−0.04	0.11*	0.14**	−0.12+	1		
8 AC Age 11 RPI	0.02	0.03	0.05	−0.01	0.06	−0.08	0.16**	1	
9 AC Age 13‐15 RPI	−0.11	−0.03	−0.09	0.08	−0.06	−0.05	−0.00	0.25***	1

*Note:* The impulsivity composite score was created by averaging the standardized scores of ATQ and Barkley's.

Abbreviations: AC = adopted child, AP = adoptive parent, ATQ = adult temperament questionnaire, Barkley's = Barkley's Adult ADHD Subscales, BP = birth parent, RPI = resistance to peer influence

* *p* < 0.05;

** *p* < 0.01;

*** *p* < 0.001.

^+^ *p* = 0.05–0.08;

### 3.3 Path Model Results

The assumptions of independence of residuals, linearity, homoscedasticity, multicollinearity, and residual normality were checked before running SEMs (syntax and output are available at https://osf.io/y39b5/?view_only=29784587ec7a4f5a9cc3d12704e5e690). The data passed all assumptions but homoscedasticity.

**Figure 1:**
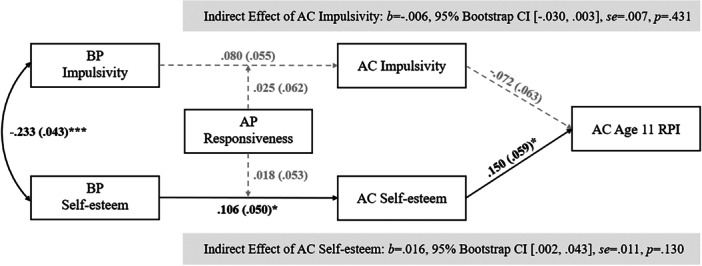
Structural equation model of birth parent impulsivity and self‐esteem, child impulsivity and self‐esteem, adoptive parent responsiveness, and adolescent RPI at age 11, with nonsignificant paths in dashed lines. *Note:* ^+^ *p* = 0.05–0.08; *p < 0.05; **p < 0.01; ***p < 0.001. All presented coefficients are standardized estimates, and the coefficients in parentheses represent standard errors. AC = adopted child, AP = adoptive parent, BP = birth parent, RPI = resistance to peer influence. AC sex, BP‐AP openness, and birth mother obstetric complications are included as covariates for model mediators; AC sex, AC age at RPI measurement, BP‐AP openness, and birth mother obstetric complications are included as covariates for outcome. The coefficients for covariates have been omitted from the figure to maintain the simplicity.

**Figure 2:**
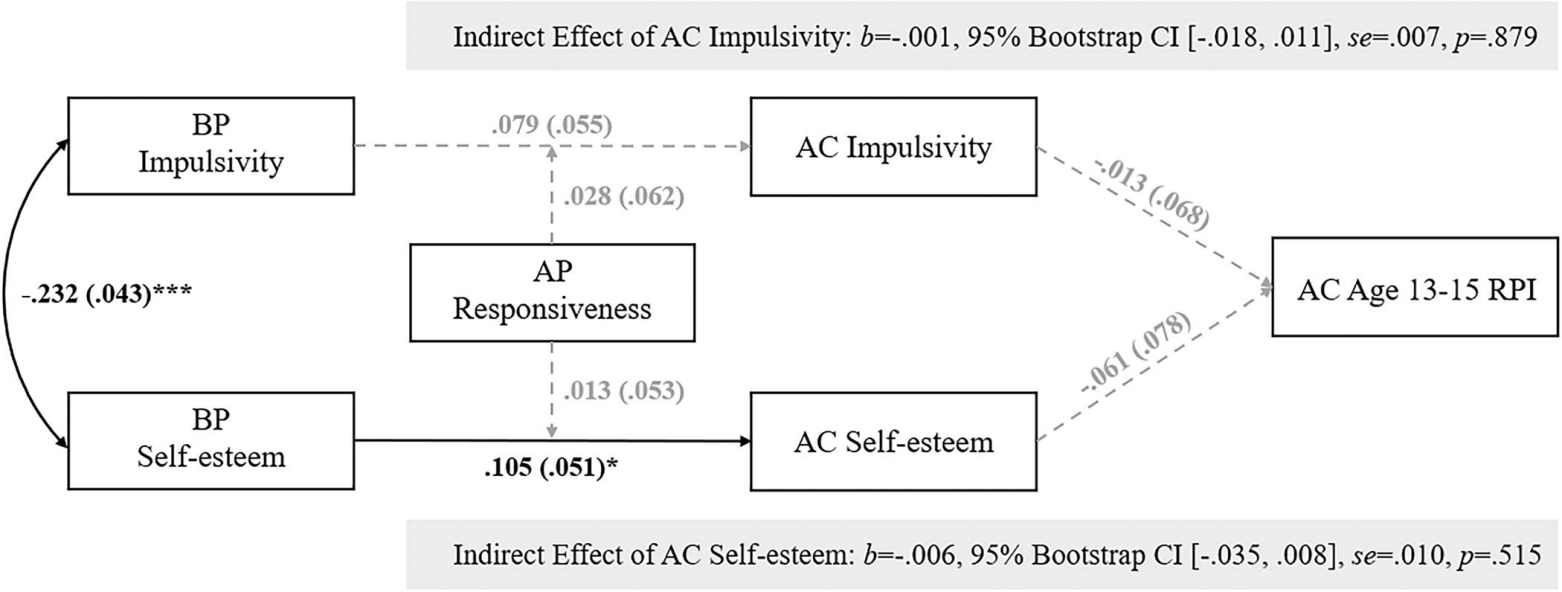
Structural equation model of birth parent impulsivity and self‐esteem, child impulsivity and self‐esteem, adoptive parent responsiveness, and adolescent RPI at age 13–15, with nonsignificant paths in dashed lines. *Note:* ^+^ *p* = 0.05–0.08; *p < 0.05; **p < 0.01; ***p < 0.001. All presented coefficients are standardized estimates, and the coefficients in parentheses represent standard errors. AC = adopted child, AP = adoptive parent, BP = birth parent, RPI = resistance to peer influence. AC sex, BP‐AP openness, and birth mother obstetric complications are included as covariates for model mediators; AC sex, AC age at RPI measurement, BP‐AP openness, and birth mother obstetric complications are included as covariates for outcome. The coefficients for covariates have been omitted from the figure to maintain the simplicity.

Both moderated mediation models for age 11 and 13‐15 RPI yielded good fit to the data, [age 11: *χ*2(10) = 11.20, *p* = 0.34; *CFI* = 0.97, *TLI* = 0.89; *RMSEA* = 0.02, *p* = 0.95; age 13–15: *χ* ^2^(12) = 20.37, *p* = 0.06; *CFI* = 0.78, *TLI* = 0.38; *RMSEA* = 0.04, *p* = 0.80]. SEMs indicated that birth parent self‐esteem positively predicted child self‐esteem at age 6–8 (*b* = 0.11, se = 0.05, *p* = 0.04), and child self‐esteem positively predicted age 11 RPI (*b* = 0.15, se = 0.06, *p* = 0.01). This indirect path was statistically significant based on the 95% Bootstrap CI (*b* = 0.02, 95% Bootstrap CI [0.002, 0.043], se = 0.01, *p* = 0.13), though the *p*‐value was not significant. Girls reported higher levels of age 11 RPI than boys (*b* = 0.20, se = 0.05, *p* < 0.001). In the age 13–15 RPI model, age 11 RPI showed moderate stability with age 13–15 RPI (*b* = 0.27, se = 0.07, *p* < 0.001); all other paths, including moderation, were not statistically significant. Standardized coefficient estimates are shown in Table 3, Figure 1 (age 11 model), and Figure 2 (age 13–15 model).

**Table 3:** Standardized coefficient estimates of SEM.

	Age 11 RPI Model				Age 13–15 RPI Model			
	*b*	se	*p*	*95% Bootstrap CI*	*b*	se	*p*	*95% Bootstrap CI*
*Outcome: AC Impulsivity*								
BP Impulsivity	0.080	0.055	0.144	[−0.031, 0.186]	0.079	0.055	0.156	[−0.032, 0.185]
BP Impulsivity*AP Responsiveness	0.025	0.062	0.680	[−0.100, 0.143]	0.028	0.062	0.651	[−0.099, 0.144]
AC Sex	−0.055	0.054	0.305	[−0.156, 0.055]	−0.053	0.053	0.324	[−0.153, 0.057]
BP‐AP Openness	0.037	0.057	0.508	[−0.072, 0.151]	0.040	0.056	0.477	[−0.069, 0.153]
BM Obstetric complications	0.039	0.048	0.416	[−0.057, 0.127]	0.038	0.048	0.423	[−0.057, 0.128]
*Outcome: AC Self‐esteem*								
BP Self‐esteem	0.106	0.050	0.036	[0.007, 0.200]	0.105	0.051	0.039	[0.004, 0.200]
BP Self‐esteem*AP Responsiveness	0.018	0.053	0.741	[−0.085, 0.121]	0.013	0.053	0.808	[−0.091, 0.114]
AC Sex	0.094	0.049	0.053	[−0.002, 0.188]	0.095	0.049	0.051	[−0.001, 0.188]
BP‐AP Openness	0.087	0.047	0.063	[−0.003, 0.180]	0.086	0.047	0.066	[−0.006, 0.179]
BM Obstetric complications	0.035	0.047	0.454	[−0.056, 0.127]	0.029	0.047	0.533	[−0.065, 0.121]
*Outcome: AC RPI*								
AC Impulsivity	−0.072	0.063	0.248	[−0.199, 0.051]	−0.013	0.068	0.852	[−0.143, 0.126]
AC Self‐esteem	0.150	0.059	0.011	[0.029, 0.264]	−0.061	0.078	0.435	[−0.212, 0.089]
BP Impulsivity → AC Impulsivity	−0.006	0.007	0.431	[−0.030, 0.003]	−0.001	0.007	0.879	[−0.018, 0.011]
BP Self‐esteem → AC Self‐esteem	0.016	0.011	0.130	[0.002, 0.043]	−0.006	0.010	0.515	[−0.035, 0.008]
AC Age	0.013	0.049	0.786	[−0.084, 0.110]	0.054	0.064	0.395	[−0.073, 0.177]
AC Sex	0.198	0.048	< 0.001	[0.103, 0.290]	0.095	0.063	0.130	[−0.031, 0.215]
BP‐AP Openness	0.020	0.057	0.727	[−0.090, 0.128]	0.036	0.056	0.523	[−0.077, 0.146]
BM Obstetric complications	−0.022	0.051	0.670	[−0.120, 0.076]	−0.014	0.058	0.812	[−0.125, 0.103]
AC Age 11 RPI	—	—	—	—	0.271	0.070	< 0.001	[0.125, 0.401]

*Note:* The models were tested using bootstrapped confidence intervals with 5000 resamples (i.e., 95% Bootstrap CI). *b* = standardized coefficient estimate; *se* = standard error.

Abbreviations: AC = adopted child, AP = adoptive parent, BM = birth mother, BP = birth parent, RPI = resistance to peer influence.

### 3.4 Sensitivity Analyses

In the main analyses, the BP impulsivity composite score consists of two subscales. Given the low correlation between subscales, models were then run separately for each subscale. Results revealed no differences from the main analyses (see Supporting Information). Additional post‐hoc sensitivity analyses requested through the review process, including 1) testing the moderating role of primary and second caregivers separately, 2) multi‐group analysis for boys and girls separately, and 3) investigating the moderation effect of parental responsiveness for all paths, are available at https://osf.io/y39b5/?view_only=29784587ec7a4f5a9cc3d12704e5e690.

## 4 Discussion

The present study aimed to investigate predictors of RPI from childhood to adolescence by testing mediating pathways of impulsivity and self‐esteem between genetic factors and RPI, and whether parental responsiveness moderated the genetic predispositions towards impulsivity and/or self‐esteem. Leveraging the adopted‐at‐birth design ruled out the potential influence of passive *r*GE that is prevalent in traditional family designs, and helped elucidate distinct roles of heritable and familial environmental factors. We found that self‐esteem, but not impulsivity, mediated heritable predispositions toward RPI (i.e., hypotheses 2 was confirmed, but hypothesis 1 was not). Further, we found no evidence that adoptive parent responsiveness moderated genetic influences on impulsivity or self‐esteem (i.e., hypotheses 3 and 4 were not confirmed). No significant paths were detected predicting adolescent age 13–15 RPI, controlling for age 11 RPI. Along with the lower correlation of self‐esteem with age 13–15 RPI (*r* = −0.00) than with age 11 RPI (*r* = 0.16; Fisher‐Z = 1.89, *p* = 0.06), findings suggest that the association between childhood self‐esteem and early and mid‐adolescent RPI may fade somewhat with time.

### 4.1 Predictors of RPI

There was only very modest stability in RPI over time, despite similar sample distributions at each time‐point, suggesting that early‐to‐mid‐adolescence is a time of substantial individual differences and nonsystematic change in RPI. Our study is the first study leveraging longitudinal data to explore the association of self‐esteem in childhood and RPI in later years, given that all previous significant associations of self‐esteem and RPI were detected in cross‐sectional studies (Bámaca and Umaña‐Taylor 2006; Stautz and Cooper 2014). The lack of association between self‐esteem and RPI at age 13–15, yet significant prediction of age 11 RPI, underscores the possibility that different factors predict early RPI versus sustained or growth in RPI across this critical developmental period for social development. One possibility is that self‐esteem constantly plays an important role in the development of RPI, but only when the two constructs are measured in closer time intervals, as both are susceptible to change during adolescence (Birkeland et al. 2012; Steinberg and Monahan 2007). In our study, however, child self‐esteem and age 13–15 RPI were measured at least 5 years apart.

### 4.2 The Mediating Role of Child Phenotypic Impulsivity and Self‐Esteem

Consistent with hypotheses 2, we detected an indirect effect of child self‐esteem, that genetic factors for self‐esteem positively predicted child self‐esteem at age 7, which in turn positively predicted RPI at age 11. This finding aligns with prior work showing that high self‐esteem is associated with greater resistance to undesired peer pressure in adolescents (Bámaca and Umaña‐Taylor 2006).

The detected mediating pathway may be partially explained by neurological mechanism. Previous research has linked genetic factors related to self‐esteem with increased activity in dorsal anterior cingulate cortex and insula, brain regions responsible for rejection sensitivity, reward, and decision making (Eisenberger et al. 2011; Izuma et al. 2018). These neurological changes may manifest on the trait level as higher self‐evaluations and greater self‐reliance when making decisions, which further supports the skills to resist peer norms and pressure (Pei et al. 2020; Pfeifer et al. 2011). No mediating pathway toward RPI at age 13–15, however, arise in current study, which can also be due to the long interval between assessment of child self‐esteem and age 13–15 RPI.

The significant indirect pathway was only detected using 95% Bootstrap CIs, but not *p*‐values. This discrepancy may be due to non‐normality of sample distribution (Preacher and Hayes 2008). While *p*‐values assumes both univariate and multivariate normality, bootstrapping does not rely on the assumption of normality when computing indirect effects (Örs Özdil and Kutlu 2019). Although our models passed the assumption check for multivariate normality for main variables, the distribution of the moderator, parental responsiveness, still remained skewed. In this case, the 95% Bootstrap CIs may provide more adequate and robust estimates.

Contrary to hypothesis 1, no evidence was found for the mediating role of child impulsivity. Whereas Stautz and Cooper (2014) found that British adolescents who were higher in negativity urgency manifested higher susceptibility to peer influence, their study was cross‐sectional. In contrast, we found that impulsivity was not related to adolescent RPI examined longitudinally. Other precursors of RPI found in longitudinal research, such as emotional autonomy and the social status of an individual's close friends (Allen et al. 2012), might be more influential.

Alternatively, it is possible that impulsivity still plays a role in the development of RPI, but the long time interval between assessments may weakened this link. It might be that impulsivity in late childhood or early adolescence can act as mediators for RPI, but earlier impulsivity cannot, as both characteristics continue to develop during the transition from childhood to adolescence (e.g., Orth et al. 2018). Future longitudinal designs with assessments having optimal reliability and validity, and closer time intervals between assessments, are essential to draw conclusions.

### 4.3 The Moderating Role of Parental Responsiveness

Our results did not detect a moderating role of parental responsiveness for genetic risks on impulsivity (hypothesis 3) or genetic influences on self‐esteem (hypothesis 4). No previous studies so far have examined the effect of parental responsiveness in moderating the genetic influences on self‐esteem, so our finding is novel. Although previous evidence indicated that parental emotional and instrumental support responding to child's needs buffered the influence of genetic risk on impulsivity and related constructs in children (Marzilli et al. 2023; Paaver et al. 2008), those findings through traditional family design might depend on the fact that parents' responsiveness shares common genetic predispositions with impulsivity and/or self‐esteem (i.e., passive *r*GE), which was ruled out from our study by using the adopted‐at‐birth design.

Additionally, parenting factors and child/adolescent outcomes are often measured at the same timepoint (i.e., cross‐sectionally) (Marzilli et al. 2023; Paaver et al. 2008), while the measures of parental responsiveness and child impulsivity/self‐esteem are at least 4 years apart in current study. This may result in insignificant findings of the moderating role of parental responsiveness, and future studies may consider collecting data in time intervals that are more closely aligned with developmental changes.

Correlation results indicated that adoptive parent's responsiveness was positively correlated with child self‐esteem, which is consistent with prior evidence on elementary school children (Kim 2022). This finding suggests that parental responsiveness may act as a promotive factor that nurtures child's self‐esteem directly (e.g., Pinquart and Gerke 2019) or may act through the evocative process of child's early manifestation of self‐esteem (e.g., Otterpohl et al. 2021), rather than acting as an environmental moderator. Future investigation of the development of RPI may need to consider these pathways of how parenting behaviors link to child phenotypes.

### 4.4 Gender Differences

Although not the focus of the investigation, we found that at age 11, girls were more likely to stand up for their own thoughts and values, rather than conforming to the norms and expectations of their peer groups, compared with boys. This finding was consistent with previous evidence (Steinberg and Monahan 2007), which argues that girls exhibit higher self‐reliance than boys in adolescence. Girls' widely acknowledged greater sensitivity to social relations might actually only apply under certain circumstances (i.e., facing peer victimization) (Calleja and Rapee 2020), rather than being translated into behavioral conformity under all conditions. Moreover, this sex difference in levels of RPI in mid‐adolescence can possibly explain the growing sex disparities in substance use and antisocial behavior after individuals entering adolescence (Cale and Lilienfeld 2002; Chen and Jacobson 2012). Boys may be less likely to resist or refuse the pressure of conduct antisocial behavior and substance use spreading in peer networks and thus become particularly predisposed to acquire such negative behaviors. Promoting the RPI skills, such as distinguishing and rejecting undesired peer norms strategically, therefore, can be a critical target for preventions and interventions addressing negative behaviors.

### 4.5 Strengths, Limitations, and Future Directions

By leveraging an adopted‐at‐birth design, we were able to elucidate the specific roles of heritable and parenting influences, by eliminating potential impacts of that parental practice is affected by the phenotypes of study (i.e., passive *r*GE). However, it is important to note that using birth parent's phenotypes is a limited measure to infer the genetic factors. The assessments of impulsivity and self‐esteem in birth parent and the child were not parallel. Also, according to multifinality in developmental science (Cicchetti and Rogosch 1996; Cloninger et al. 2019), a child who carries behavioral impulsivity‐related genetic factors from their birth parents may finally show emotional impulsive symptoms, inattention, or other externalizing problems. Future studies may consider using polygenic risk scores based on large‐scale genome‐wide association studies, to obtain a more robust proxy of genetic factors.

The current study is a multi‐method study consisting of multiple forms and informants of measures (i.e., parent report on the child, child self‐report, observation), and hence avoids the inflation of effects due to shared method variance, making the inferences of research more conservative. The assessment of parental responsiveness via interviewer ratings on a standardized measure after home visits is considered a strength overall, as it is a more objective measure of responsiveness than parent self‐report, to minimize social desirability. However, observational measures of specific moments in a family's life can also have reduced sensitivity in capturing the complexities of responsiveness in real life. It should also be noted that the alphas for the responsiveness scale were lower than optimal, and that the scale was extremely negatively skewed, which suggested a ceiling effect of responsiveness in adoptive parents. Future research may want to test the roles of parenting in child RPI in wider populations, such as non‐adoptive families and families more diverse in socioeconomic status.

## 5 Conclusion

Overall, by using the adopted‐at‐birth sample, this study examined the specific roles of heritable and parenting factors and extends our understanding of separate as well as joint roles multi‐layered factors (e.g., genetic factors, individual traits, familial environment) in the levels of skills to resist undesired peer pressure. The results only supported our hypothesis 2, that child self‐esteem mediated the association of genetic factors for self‐esteem and RPI in early adolescence. Child impulsivity did not reveal any mediating pathways, nor did parental responsiveness moderate genetic influences on impulsivity and self‐esteem. Future studies should consider creating more robust indexes of genetic factors for child phenotypes, and exploring other individual or environmental factors in the developmental trajectories of RPI (Allen et al. 2012).

## Ethics Statement

The data collection used in the current study was approved by the Institutional Review Board at the University of Oregon (IRB#04262013.034; IRB#03042014.001; IRB#04262013.035; IRB#08082016.007), George Washington University (IRB#041119), Penn State University (STUDY00007603), and use of these data for the current study was approved by Purdue University (IRB‐2022‐522).

## Conflicts of Interest

The authors declare no conflicts of interest.

## Supporting information

JOA_Supplementary_Materials_060525.

## Data Availability

Data supporting the findings of this study are available upon reasonable request to the study PIs (Ganiban, Leve, and Neiderhiser). All data preparation and analytic code and results are available via the tech report available at https://osf.io/y39b5/?view_only=29784587ec7a4f5a9cc3d12704e5e690.
