# Supplementary material for: A Moderated Mediation Model to Predict Adolescent Resistance to Peer Influence: Evidence From an Adoption Study
Source: J Adolesc. 2025 Jul 13;97(7):1965–77. doi: 10.1002/jad.70016 (PMC12493014; doi:10.1002/jad.70016)
Supplement: Supplementary file 1 — JOA_Supplementary_Materials_060525. [file JAD-97-1965-s001.docx]

**Supplementary Materials**

Table S1. *Bivariate correlation results of main variables and covariates*

|  | 1 | 2 | 3 | 4 | 5 | 6 | 7 | 8 | 9 | 10 | 11 | 12 | 13 | 14 |
| --- | --- | --- | --- | --- | --- | --- | --- | --- | --- | --- | --- | --- | --- | --- |
| 1 BP Impulsivity (ATQ) | 1 |  |  |  |  |  |  |  |  |  |  |  |  |  |
| 2 BP Impulsivity (Barkley’s) | .21*** | 1 |  |  |  |  |  |  |  |  |  |  |  |  |
| 3 BP Impulsivity (composite) | .80*** | .82*** | 1 |  |  |  |  |  |  |  |  |  |  |  |
| 4 BP Self-esteem | -.16*** | -.20*** | -.23*** | 1 |  |  |  |  |  |  |  |  |  |  |
| 5 AP Responsiveness | .05 | .00 | .04 | .08^+^ | 1 |  |  |  |  |  |  |  |  |  |
| 6 AC Impulsivity | -.00 | .10^+^ | .08 | -.13* | -.09 | 1 |  |  |  |  |  |  |  |  |
| 7 AC Self-esteem | -.10 | .00 | -.04 | .11* | .14** | -.12^+^ | 1 |  |  |  |  |  |  |  |
| 8 AC Age 11 RPI | .02 | .03 | .05 | -.01 | .06 | -.08 | .16** | 1 |  |  |  |  |  |  |
| 9 AC Age 13-15 RPI | -.11 | -.03 | -.09 | .08 | -.06 | -.05 | -.00 | .25*** | 1 |  |  |  |  |  |
| 10 AC Age at first RPI measure | -.02 | -.01 | -.02 | -.05 | .02 | -.01 | -.01 | .02 | .06 | 1 |  |  |  |  |
| 11 AC Age at second RPI measure | .00 | -.02 | -.01 | .05 | -.16** | -.04 | .00 | -.08 | .05 | -.41* | 1 |  |  |  |
| 12 AC Sex | .11 | -.03 | .02 | -.03 | -.00 | -.05 | .09 | .21*** | .14* | .02 | .05 | 1 |  |  |
| 13 BP-AP Openness | -.02 | .10* | .04 | .05 | .01 | .05 | .08 | .02 | .03 | .01 | -.01 | -.06 | 1 |  |
| 14 BM Prenatal complications | .08 | .19*** | .19*** | -.18*** | -.00 | .05 | .01 | -.01 | -.04 | .01 | .04 | -.00 | -.03 | 1 |

*Note:* ^+^*p* = .05-.08; ^*^*p* < .05; ^**^*p* < .01; ^***^*p* < .001. BP = birth parent; BM = birth mother; AP = adoptive parent; AC = adopted child. RPI = resistance to peer influence. ATQ = Adult Temperament Questionnaire; Barkley’s = Barkley’s Adult ADHD Subscales; the impulsivity composite score was created by averaging the standardized scores of ATQ and Barkley’s.

Table S2. *Standardized coefficient estimates of SEM (Birth parent impulsivity indexed by the Adult Temperament Questionnaire)*

|  | **Age 11 RPI Model** | | | |  | **Age 13-15 RPI Model** | | | |
| --- | --- | --- | --- | --- | --- | --- | --- | --- | --- |
|  | ***b*** | ***se*** | ***p*** | ***95% Bootstrap CI*** |  | ***b*** | ***se*** | ***p*** | ***95% Bootstrap CI*** |
| ***Outcome: AC Impulsivity*** |  |  |  |  |  |  |  |  |  |
| BP Impulsivity | .021 | .058 | .718 | [-.099, .133] |  | .019 | .059 | .751 | [-.105, .131] |
| BP Impulsivity*AP Responsiveness | -.021 | .067 | .759 | [-.160, .103] |  | -.013 | .066 | .843 | [-.151, .112] |
| AC Sex | -.060 | .054 | .262 | [-.161, .048] |  | -.057 | .054 | .286 | [-.158, .050] |
| BP-AP Openness | .038 | .056 | .498 | [-.071, .150] |  | .040 | .056 | .472 | [-.068, .152] |
| BM Obstetric complications | .054 | .047 | .256 | [-.043, .142] |  | .053 | .047 | .262 | [-.044, .141] |
| ***Outcome: AC Self-esteem*** |  |  |  |  |  |  |  |  |  |
| BP Self-esteem | .105 | .050 | .037 | [.007, .201] |  | .104 | .051 | .040 | [.004, .200] |
| BP Self-esteem*AP Responsiveness | .017 | .053 | .745 | [-.086, .120] |  | .013 | .053 | .806 | [-.090, .114] |
| AC Sex | .094 | .049 | .054 | [-.002, .187] |  | .095 | .049 | .051 | [-.001, .188] |
| BP-AP Openness | .087 | .047 | .063 | [-.003, .180] |  | .087 | .047 | .066 | [-.006, .179] |
| BM Obstetric complications | .036 | .047 | .453 | [-.056, .128] |  | .029 | .047 | .534 | [-.065, .121] |
| ***Outcome: AC RPI*** |  |  |  |  |  |  |  |  |  |
| AC Impulsivity | -.068 | .063 | .278 | [-.194, .055] |  | -.022 | .067 | .748 | [-.152, .116] |
| AC Self-esteem | .148 | .059 | .013 | [.028, .262] |  | -.066 | .076 | .387 | [-.213, .082] |
| BP Impulsivity 🡪 AC Impulsivity | -.001 | .005 | .794 | [-.019, .005] |  | .000 | .004 | .926 | [-.014, .006] |
| BP Self-esteem 🡪 AC Self-esteem | .016 | .010 | .134 | [.001, 043] |  | -.007 | .010 | .475 | [-.035, .007] |
| AC Age | .012 | .050 | .805 | [-.087, .109] |  | .051 | .064 | .426 | [-.075, .175] |
| AC Sex | .197 | .049 | <.001 | [.100, .293] |  | .108 | .062 | .082 | [-.019, .226] |
| BP-AP Openness | .026 | .056 | .649 | [-.082, .134] |  | .033 | .055 | .549 | [-.076, .141] |
| BM Obstetric complications | -.013 | .050 | .799 | [-.111, .082] |  | -.021 | .056 | .714 | [-.128, .091] |
| AC Age 11 RPI | -- | -- | -- | -- |  | .266 | .070 | <.001 | [.123, .398] |

*Note:* *b* = standardized coefficient estimate; *se* = standard error. BP = birth parent; BM = birth mother; AP = adoptive parent; AC = adopted child. RPI = resistance to peer influence.


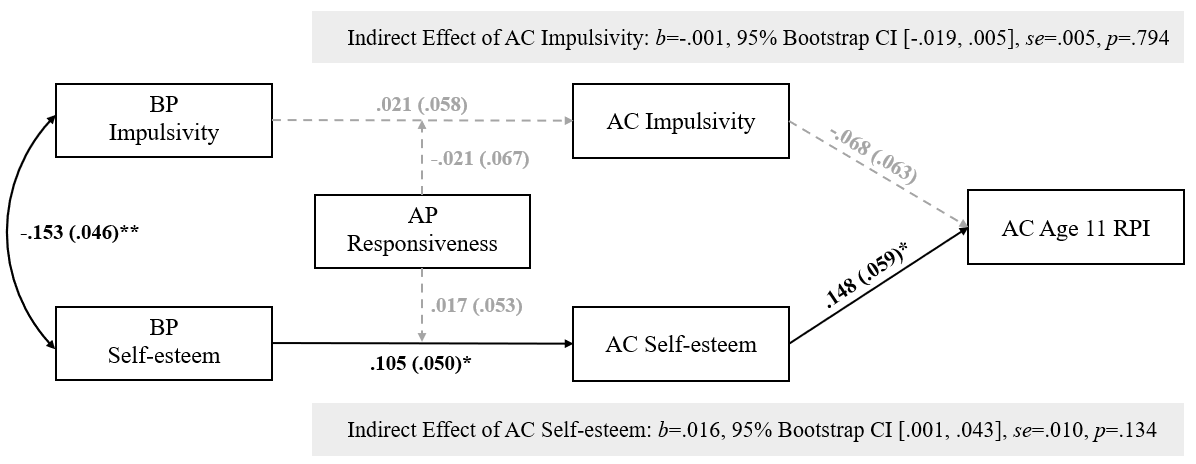


Figure S1. Structural equation model of birth parent impulsivity (indexed by the Adult Temperament Questionnaire) and self-esteem, child impulsivity and self-esteem, adoptive parent responsiveness, and adolescent RPI at age 11.

*Note:* ^+^*p* = .05-.08; ^*^*p* < .05; ^**^*p* < .01; ^***^*p* < .001. All presented coefficients are standardized estimates, and the coefficients in parentheses represent standard errors. BP = birth parent; AP = adoptive parent; AC = adopted child. RPI = resistance to peer influence. AC sex, BP-AP openness, and birth mother obstetric complications are included as covariates for model mediators; AC sex, AC age at RPI measurement, BP-AP openness, and birth mother obstetric complications are included as covariates for outcome. The coefficients for covariates have been omitted from the figure to maintain the simplicity.


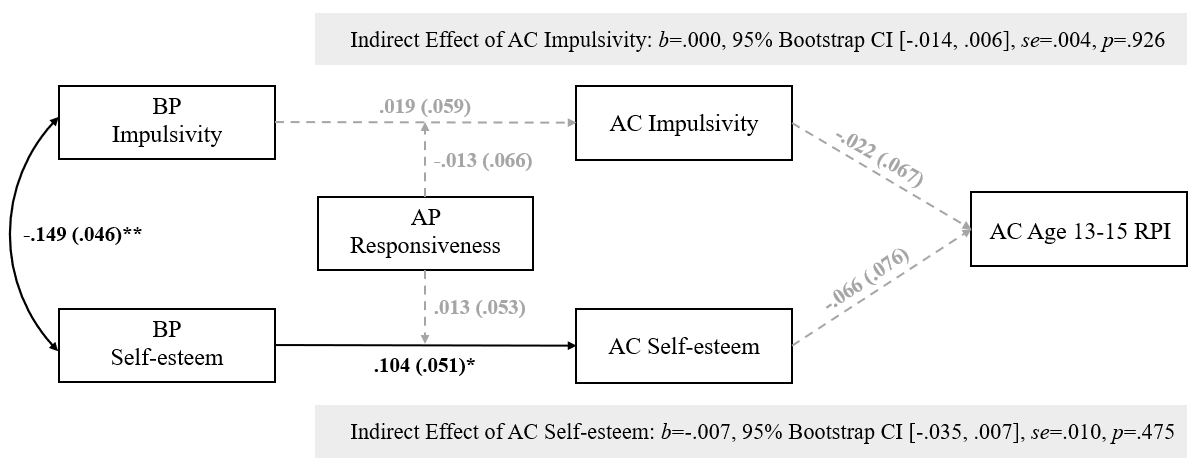


Figure S2. Structural equation model of birth parent impulsivity (indexed by the Adult Temperament Questionnaire) and self-esteem, child impulsivity and self-esteem, adoptive parent responsiveness, and adolescent RPI at age 13-15.

*Note:* ^+^*p* = .05-.08; ^*^*p* < .05; ^**^*p* < .01; ^***^*p* < .001. All presented coefficients are standardized estimates, and the coefficients in parentheses represent standard errors. BP = birth parent; AP = adoptive parent; AC = adopted child. RPI = resistance to peer influence. AC sex, BP-AP openness, and birth mother obstetric complications are included as covariates for model mediators; AC sex, AC age at RPI measurement, BP-AP openness, and birth mother obstetric complications are included as covariates for outcome. The coefficients for covariates have been omitted from the figure to maintain the simplicity.

Table S3. *Standardized coefficient estimates of SEM (Birth parent impulsivity indexed by the Barkley’s Adult ADHD Scales)*

|  | **Age 11 RPI Model** | | | |  | **Age 13-15 RPI Model** | | | |
| --- | --- | --- | --- | --- | --- | --- | --- | --- | --- |
|  | ***b*** | ***se*** | ***p*** | ***95% Bootstrap CI*** |  | ***b*** | ***se*** | ***p*** | ***95% Bootstrap CI*** |
| ***Outcome: AC Impulsivity*** |  |  |  |  |  |  |  |  |  |
| BP Impulsivity | .098 | .058 | .093 | [-.020, .207] |  | .096 | .058 | .100 | [-.024, .205] |
| BP Impulsivity*AP Responsiveness | .069 | .065 | .288 | [-.063, .191] |  | .071 | .065 | .273 | [-.060, .192] |
| AC Sex | -.049 | .053 | .349 | [-.148, .059] |  | -.047 | .053 | .367 | [-.146, .060] |
| BP-AP Openness | .034 | .057 | .554 | [-.076, .147] |  | .036 | .057 | .523 | [-.074, .149] |
| BM Obstetric complications | .034 | .048 | .473 | [-.061, .125] |  | .034 | .048 | .482 | [-.062, .123] |
| ***Outcome: AC Self-esteem*** |  |  |  |  |  |  |  |  |  |
| BP Self-esteem | .105 | .050 | .037 | [.007, .200] |  | .104 | .051 | .040 | [.004, .200] |
| BP Self-esteem*AP Responsiveness | .017 | .053 | .748 | [-.086, .119] |  | .013 | .053 | .811 | [-.090, .114] |
| AC Sex | .094 | .049 | .053 | [-.002, .187] |  | .095 | .049 | .051 | [-.001, .188] |
| BP-AP Openness | .087 | .047 | .064 | [-.003, .180] |  | .086 | .047 | .066 | [-.006, .179] |
| BM Obstetric complications | .035 | .047 | .455 | [-.056, .128] |  | .029 | .047 | .534 | [-.064, .121] |
| ***Outcome: AC RPI*** |  |  |  |  |  |  |  |  |  |
| AC Impulsivity | -.073 | .063 | .250 | [-.201, .051] |  | -.009 | .068 | .898 | [-.140, .128] |
| AC Self-esteem | .149 | .059 | .011 | [.029, .261] |  | -.059 | .077 | .444 | [-.209, .090] |
| BP Impulsivity 🡪 AC Impulsivity | -.007 | .009 | .416 | [-.034, .003] |  | -.001 | .008 | .915 | [-.019, .014] |
| BP Self-esteem 🡪 AC Self-esteem | .016 | .010 | .132 | [.001, 043] |  | -.006 | .010 | .522 | [-.034, .008] |
| AC Age | .012 | .049 | .808 | [-.086, .109] |  | .054 | .064 | .395 | [-.072, .176] |
| AC Sex | .201 | .048 | <.001 | [.106, .295] |  | .101 | .062 | .105 | [-.022, .221] |
| BP-AP Openness | .016 | .058 | .786 | [-.095, .127] |  | .035 | .057 | .546 | [-.080, .144] |
| BM Obstetric complications | -.023 | .051 | .659 | [-.124, .076] |  | -.024 | .058 | .676 | [-.136, .092] |
| AC Age 11 RPI | -- | -- | -- | -- |  | .266 | .069 | <.001 | [.119, .394] |

*Note:* *b* = standardized coefficient estimate; *se* = standard error. BP = birth parent; BM = birth mother; AP = adoptive parent; AC = adopted child. RPI = resistance to peer influence.


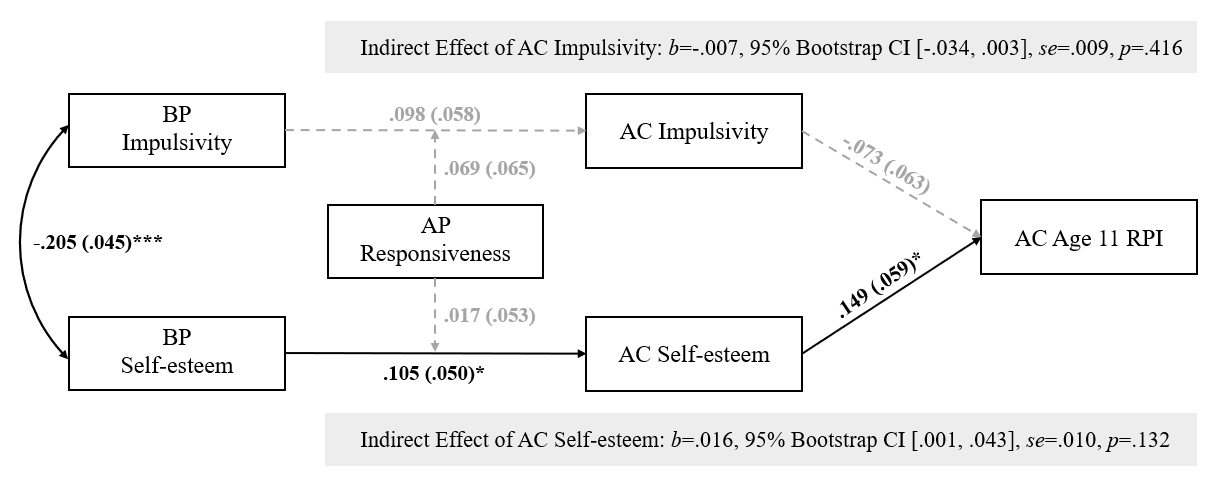


Figure S3. Structural equation model of birth parent impulsivity (indexed by the Barkley’s Adult ADHD Scales) and self-esteem, child impulsivity and self-esteem, adoptive parent responsiveness, and adolescent RPI at age 11.

*Note:* ^+^*p* = .05-.08; ^*^*p* < .05; ^**^*p* < .01; ^***^*p* < .001. All presented coefficients are standardized estimates, and the coefficients in parentheses represent standard errors. BP = birth parent; AP = adoptive parent; AC = adopted child. RPI = resistance to peer influence. AC sex, BP-AP openness, and birth mother obstetric complications are included as covariates for model mediators; AC sex, AC age at RPI measurement, BP-AP openness, and birth mother obstetric complications are included as covariates for outcome. The coefficients for covariates have been omitted from the figure to maintain the simplicity.


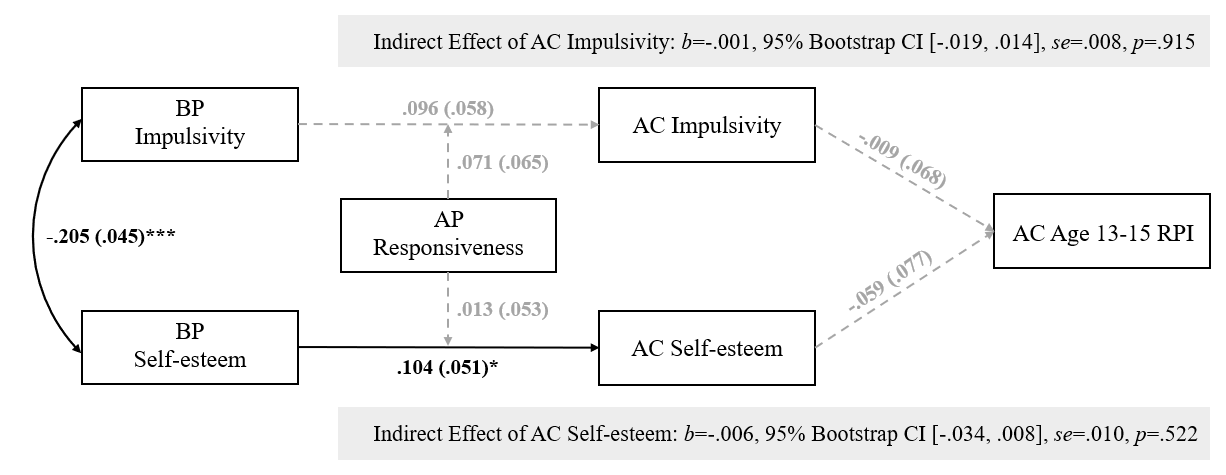


Figure S4. Structural equation model of birth parent impulsivity (indexed by the Barkley’s Adult ADHD Scales) and self-esteem, child impulsivity and self-esteem, adoptive parent responsiveness, and adolescent RPI at age 13-15.

*Note:* ^+^*p* = .05-.08; ^*^*p* < .05; ^**^*p* < .01; ^***^*p* < .001. All presented coefficients are standardized estimates, and the coefficients in parentheses represent standard errors. BP = birth parent; AP = adoptive parent; AC = adopted child. RPI = resistance to peer influence. AC sex, BP-AP openness, and birth mother obstetric complications are included as covariates for model mediators; AC sex, AC age at RPI measurement, BP-AP openness, and birth mother obstetric complications are included as covariates for outcome. The coefficients for covariates have been omitted from the figure to maintain the simplicity.
